# Supplementary material for: Impaired HA-specific T follicular helper cell and antibody responses to influenza vaccination are linked to inflammation in humans
Source: eLife. 2021 Nov 2;10:e70554. doi: 10.7554/eLife.70554 (PMC8562996; doi:10.7554/eLife.70554)
Supplement: Transparent reporting form [file elife-70554-transrepform1.docx]

***eLife’s* transparent reporting form**

We encourage authors to provide detailed information *within their submission* to facilitate the interpretation and replication of experiments. Authors can upload supporting documentation to indicate the use of appropriate reporting guidelines for health-related research (see [EQUATOR Network](http://www.equator-network.org/%20)), life science research (see the [BioSharing Information Resource](https://biosharing.org/)), or the [ARRIVE guidelines](http://www.plosbiology.org/article/info:doi/10.1371/journal.pbio.1000412) for reporting work involving animal research. Where applicable, authors should refer to any relevant reporting standards documents in this form.

If you have any questions, please consult our Journal Policies and/or contact us: [editorial@elifesciences.org](mailto:editorial@elifesciences.org).

**Sample-size estimation**

- You should state whether an appropriate sample size was computed when the study was being designed
- You should state the statistical method of sample size computation and any required assumptions
- If no explicit power analysis was used, you should describe how you decided what sample (replicate) size (number) to use

Please outline where this information can be found within the submission (e.g., sections or figure legends), or explain why this information doesn’t apply to your submission:

Our cohorts are outlined in the Materials and Methods section under “*Human cohorts*”.

Being the first study of its kind to measure antigen-specific T follicular helper cells using tetramers on *HLADR*0701* or *HLADR*1101* individuals, it was not possible to perform power calculations prior to conducting the cohort. We chose to select 16-18 individuals per age group in order be able reasonably capture a sense of the variability within the immune parameters we measured.

We performed a second independent, replication cohort to powered confirm the results from cohort 1 with 21 individuals per age group.

**Replicates**

- You should report how often each experiment was performed
- You should include a definition of biological versus technical replication
- The data obtained should be provided and sufficient information should be provided to indicate the number of independent biological and/or technical replicates
- If you encountered any outliers, you should describe how these were handled
- Criteria for exclusion/inclusion of data should be clearly stated
- High-throughput sequence data should be uploaded before submission, with a private link for reviewers provided (these are available from both GEO and ArrayExpress)

Please outline where this information can be found within the submission (e.g., sections or figure legends), or explain why this information doesn’t apply to your submission:

In all figure legends, and where appropriate in the results text, we have included sample sizes used for comparison. We performed two independent cohorts, and have focused our analysis of phenotypes that occurred consistently across these two cohorts. In most instances, each cohort is reported separately, and where data are pooled this is explicitly stated in the text and figure legends. All replication is biological rather than technical replication, which we have made clear by describing results as “n=x# of individuals”. No outliers were removed for immune parameter datasets.

We have explicitly stated why and when samples were removed from RNA sequencing analysis (i.e. during quality control screening or in order to match samples at both day 0 and day 7). We have described in the methods how we have performed batch correction to remove unwanted variation (see Methods “RNA sequencing and data processing”). Our high-throughput sequencing data has been made available to reviewers and can be access via the following GEO token GSE176447: <https://www.ncbi.nlm.nih.gov/geo/query/acc.cgi?acc=GSE176447>

Enter token qvudskmernkjhiz

**Statistical reporting**

- Statistical analysis methods should be described and justified
- Raw data should be presented in figures whenever informative to do so (typically when N per group is less than 10)
- For each experiment, you should identify the statistical tests used, exact values of N, definitions of center, methods of multiple test correction, and dispersion and precision measures (e.g., mean, median, SD, SEM, confidence intervals; and, for the major substantive results, a measure of effect size (e.g., Pearson's r, Cohen's d)
- Report exact p-values wherever possible alongside the summary statistics and 95% confidence intervals. These should be reported for all key questions and not only when the p-value is less than 0.05.

Please outline where this information can be found within the submission (e.g., sections or figure legends), or explain why this information doesn’t apply to your submission:

Statistical methods and tests are described throughout in figure legends and Materials and Methods sections. Except where otherwise indicated, all analysis methods assumed non-parametric data.

Raw data is presented throughout as dots overlaid on violin plots. Where data is summarized into heatmap format, we have included the significant/informative variables either subsequently in the figure (e.g. Figure 2), or in supplementary data (e.g. Figure S7). Median is used as the summary statistic throughout.

In each figure legend and in Materials and Methods sections we have outlined which statistical tests were used, and when and where we have performed false discovery rate (Benjamini-Hochberg) or Dunn’s test adjustment of p-values.

Correlation analysis was performed using Spearman’s correlation throughout, and Rho and p-values are reported for each comparison.

(For large datasets, or papers with a very large number of statistical tests, you may upload a single table file with tests, Ns, etc., with reference to sections in the manuscript.)

**Group allocation**

- Indicate how samples were allocated into experimental groups (in the case of clinical studies, please specify allocation to treatment method); if randomization was used, please also state if restricted randomization was applied
- Indicate if masking was used during group allocation, data collection and/or data analysis

Please outline where this information can be found within the submission (e.g., sections or figure legends), or explain why this information doesn’t apply to your submission:

Participants were recruited to the vaccination studies on the basis of age and HLADR genotype. Efforts were taken to control for gender across cohorts.

Investigators were blinded to sample age and identifying information during group allocation, sample collection, and immunological measures.

For NGS sequencing, 3 young and 3 old samples were sequenced per flow cell wherever possible to reduce batch effects.

**Additional data files (“source data”)**

- We encourage you to upload relevant additional data files, such as numerical data that are represented as a graph in a figure, or as a summary table
- Where provided, these should be in the most useful format, and they can be uploaded as “Source data” files linked to a main figure or table
- Include model definition files including the full list of parameters used
- Include code used for data analysis (e.g., R, MatLab)
- Avoid stating that data files are “available upon request”

Please indicate the figures or tables for which source data files have been provided:

Source data and R code will be published on GitHub after manuscript is accepted and linked to the article, and sequencing data will be made available on GEO.
